# Topological Regulation of the Bioactive Conformation of a Disulfide-Rich Peptide, Heat-Stable Enterotoxin

**DOI:** 10.3390/molecules25204798

**Published:** 2020-10-21

**Authors:** Shigeru Shimamoto, Mayu Fukutsuji, Toi Osumi, Masaya Goto, Hiroshi Toyoda, Yuji Hidaka

**Affiliations:** Faculty of Science and Engineering, Kindai University, 3-4-1 Kowakae, Higashi-Osaka, Osaka 577-8502, Japan; mayuchibiii@gmail.com (M.F.); css21211vctl@gmail.com (T.O.); tf.rongu.800@gmail.com (M.G.); turarakida@gmail.com (H.T.)

**Keywords:** disulfide, enterotoxin, folding, guanylyl cyclase, topological

## Abstract

Heat-stable enterotoxin (ST_a_) produced by enterotoxigenic *E. coli* causes acute diarrhea and also can be used as a specific probe for colorectal cancer cells. ST_a_ contains three intra-molecular disulfide bonds (C1–C4, C2–C5, and C3–C6 connectivity). The chemical synthesis of ST_a_ provided not only the native type of ST_a_ but also a topological isomer that had the native disulfide pairings. Interestingly, the activity of the topological isomer was approximately 1/10–1/2 that of the native ST_a_. To further investigate the bioactive conformation of this molecule and the regulation of disulfide-coupled folding during its chemical syntheses, we examined the folding mechanism of ST_a_ that occurs during its chemical synthesis. The folding intermediate of ST_a_ with two disulfide bonds (C1–C4 and C3–C6) and two Cys(Acm) residues, the precursor peptide, was treated with iodine to produce a third disulfide bond under several conditions. The topological isomer was predominantly produced under all conditions tested, along with trace amounts of the native type of ST_a_. In addition, NMR measurements indicated that the topological isomer has a left-handed spiral structure similar to that of the precursor peptide, while the native type of ST_a_ had a right-handed spiral structure. These results indicate that the order of the regioselective formation of disulfide bonds is important for the regulation of the final conformation of disulfide-rich peptides in chemical synthesis.

## 1. Introduction

Disulfide bond formation is a post-translational modification that plays an important role in the stabilization of the native conformation of numerous peptides and proteins [1,2]. The correct disulfide pairings are typically required for the expression of biological activity. Disulfide-containing peptides and proteins are ideal models for studies of protein folding, since their folding intermediates can be observed, trapped, and separated by HPLC during the refolding reaction [3,4]. In addition, a chemical procedure for the regioselective formation of disulfide bonds make it possible to regulate and analyze the structures of the folding intermediates that frequently contain mis-bridged (non-native) or native disulfide bonds [5].

**Figure 1:**
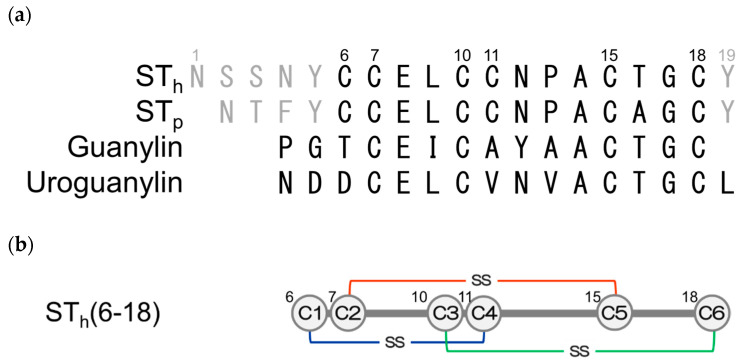
(**a**) Amino acid sequences and disulfide linkages of ST_h_ and ST_p_ produced by a human and a porcine strain of enterotoxigenic *E. coli*, respectively. (**b**) The schematic drawing of ST_h_(6–18). The Cys6, Cys7, Cys10, Cys11, Cys15, and Cys18 residues were represented by C1, C2, C3, C4, C5, and C6, respectively.

Heat-stable enterotoxin (ST_a_) produced by enterotoxigenic *E. coli* is known as an exogenous ligand of the intestinal membrane receptor guanylyl cyclase-C (GC-C) and stimulates the secretion of chloride ions via the activation of cystic fibrosis transmembrane conductance regulator as well as the endogenous ligands, uroguanylin and guanylin, of the same receptor, GC-C [6,7]. These peptide hormones contain two disulfide bonds at positions similar to those for ST_a_, which is crucial for biological activity, and they have been reported to be representative peptides that can be chemically prepared by disulfide coupled folding [8,9]. ST_a_ is further divided into ST_h_ and ST_p_ derived from a human and a porcine strain of enterotoxigenic *E. coli*, respectively [10,11], and the toxic core region consists of 13 amino acid residues including six Cys residues that form intra-molecular disulfide bonds, as shown in Figure 1 [8,9,12]. The reduced form of ST_a_ spontaneously folds into only the native conformation with the correct pairings of their disulfide bonds (C1–C4, C2–C5, and C3–C6) by air-oxidation without the need for any mercapto-reagents, such as glutathione, indicating that the amino acid sequence of ST_a_ carries sufficient information to allow the molecule to fold only into the native conformation. Thus, ST_a_ possesses a rigid conformation for a small peptide, and therefore, it would be a good model for studies of disulfide-coupled peptide and protein folding.

In our previous studies involving the chemical synthesis of ST_a_ peptides, ST_a_ was synthesized as not only the native type of ST_a_ but also as the topological isomer with native disulfide pairings formed by the stepwise formation of disulfide bonds, although only the native type of ST_a_ was produced by the simultaneous formation of three disulfide bonds using air-oxidation. Interestingly, the toxic activity of the topological isomer was still 1/10–1/2 that of the native ST_a_ [8]. The topological isomer was predominantly produced during the regioselective formation of the disulfide bonds, regardless of the order of the formation of the disulfide bridges. It is particularly noteworthy that only the topological isomer was obtained when the C2–C5 disulfide bond was formed by I_2_-oxidation at the final step [8,9]. The topological isomer is stable in the usual buffers or organic solvents without mercapto-reagents, although the isomer is immediately converted into the native conformation of ST_a_ in the presence of thiol reagents such as mercaptoethanol. These observations suggest that the topological isomer is a kinetically trapped product that is produced during the chemical synthesis, and that regioselective disulfide formation provides a product that has a different conformation from the native peptide in spite of the fact that it contains the same disulfide bonds.

Thus, the regioselective formation of the disulfide bonds of the ST_a_ peptide produces not only the topological isomer but the native type of ST_a_ as well, thus providing an ideal model for studies of peptide and protein folding that occur during chemical synthesis. In addition, it should be noted that it might not be possible to synthesize the native type of a target peptide or a protein the stepwise formation of disulfide bonds because of the production of topological isomers [13,14]. Therefore, to elucidate the mechanism responsible for the folding of the topological isomer and the native form of peptides that occur during the chemical synthesis, the precursor peptide of the topological isomer of ST_a_ was treated with iodine to form the third disulfide bond under several sets of conditions. In addition, NMR measurements of the precursor and the topological isomer were also carried out to obtain structural information on peptide and protein folding that occurs during the chemical procedures.

## 2. Results

### 2.1. Preparation of the Precursor Peptide with Two Disulfide Bonds and 2 × Cys(Acm) and the Topological Isomer

We previously reported that the topological isomer of ST_h_(6–18) was unexpectedly synthesized by stepwise regioselective disulfide formation, regardless of the order of the formation of three disulfide pairings [8]. Interestingly, only the topological isomer was produced in the case of the I_2_-oxidation of a precursor peptide that contains two Cys(Acm) residues at the C2 and C5 positions and two disulfide bonds (C1–C4 and C3–C6 connectivity). Therefore, the precursor peptide, [Cys(Acm)^7,15^,2SS]-ST_h_(6–18), was used to investigate the folding mechanism of ST_a_ in the chemical synthesis and chemically synthesized by the Boc solid phase method (Figure S1).

After deblocking the protected peptide resins with HF, the disulfide bonds were randomly formed by air-oxidation. The target precursor peptide with two disulfide bonds at C1–C4 and C3–C6 and 2 × Cys(Acm) was the major product and was purified by RP-HPLC, as shown in Figures S2A and S3. The purified precursor peptide was further treated with iodine under the standard condition using 50% MeOH to provide the topological isomer (Figure S2C), as previously described [8].

**Figure 2:**
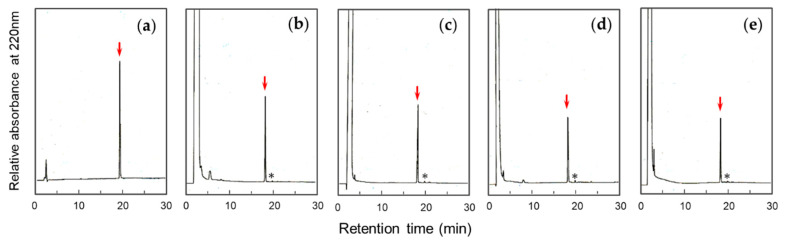
RP-HPLC profiles of (**a**) Acm_2_-precursor peptide and the reaction solutions after I_2_-oxidation in (**b**) 50% AcOH, (**c**) 50% MeOH/0.1 M HCl, (**d**) 50% *i*-PrOH/0.1 M HCl, and (**e**) 80% *i*-PrOH/0.1 M HCl with a linear gradient from 5 to 50% CH_3_CN in 45 min (l.0%/min). The asterisks (*) are the position of the expected retention time of the native type ST_h_(6–18).

In ordinary conditions of I_2_-oxidation, only a single peak was observed with the same relative intensity of that of the precursor peptide on HPLC, as shown in Figure 2a–c. The retention time of this peak was obviously different from that of native form of ST_h_(6–18). To estimate the secondary structure of the topological isomer of ST_h_(6–18) in this peak fraction, circular dichroism (CD) spectra were obtained and compared with that of the native form (Figure S5). The spectra of the peptide in Figure 2b,c showed a smaller ellipiticity at around 200 nm compared to that of the native form, suggesting that the ST_h_(6–18) peptide obtained by the present methods is the topological isomer that has a different structure from that of the native form. Thus, the topological isomer is only produced during chemical synthesis by the regioselective method with no traces of the native type ST_a_ being observed.

### 2.2. Topological Selection for the Formation of the Third Disulfide Bond Using I_2_-Oxidation

The regioselective formation of disulfide bonds in peptide syntheses usually use a combination of air-oxidation in the first step and I_2_-oxidation in the second step to avoid disulfide exchange reactions. Air-oxidation is typically carried out in an aqueous buffer at a slightly alkaline pH. However, because I_2_ is soluble in certain organic solvents, such as methanol, the reaction can be carried out in an organic solvent. The velocity and yield of disulfide bond formation are dramatically affected by the type of organic solvent [15]. Therefore, to investigate the disulfide-coupled folding of peptides and proteins that occur during chemical synthesis, solvents (AcOH, methanol, *i*-PrOH, and tetrahydrofuran) were investigated for the topological selection of ST_a_. Disulfide bond formation proceeded simultaneously with the release of the Acm groups by I_2_-oxidation. In all acidic conditions investigated for I_2_-oxidation, the topological isomer was predominantly produced with only trace amounts of the native type of ST_a_, as shown in Figure 2. These results suggest that the formation of the topological isomer depends on the conformation of the precursor peptide, suggesting that the local conformation is stabilized by hydrogen bonds and/or the bulkiness of the molecular conformation at the transitional states are important for the topological selection of ST_a_ folding to occur during chemical synthesis. Actually, these solvents provide lower permittivity for the chemical reaction and induce hydrogen bond formation under the acidic conditions as used in this study.

**Figure 3:**
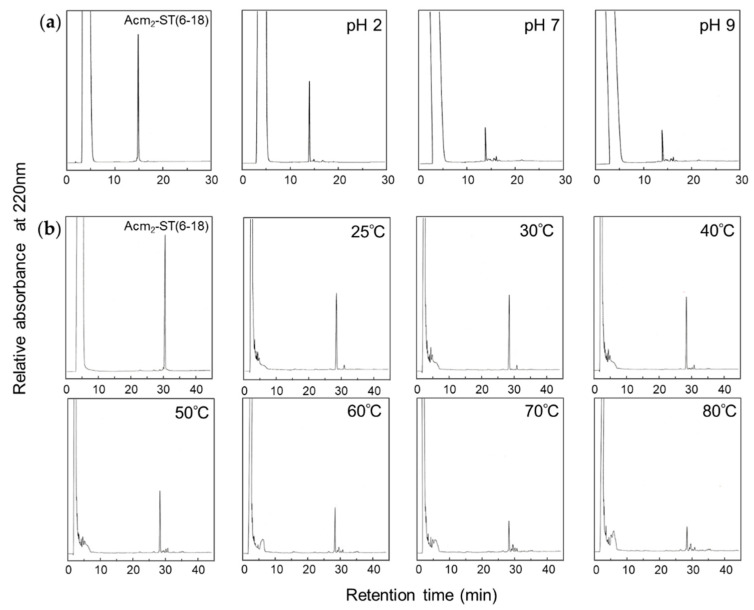
(**a**) RP-HPLC profiles of Acm_2_-precursor peptide and the reaction solutions after I_2_-oxidation in 50% MeOH at pH 2, pH 7, and pH 9 with a linear gradient from 10 to 50% CH_3_CN in 40 min (l.0%/min). (**b**) RP-HPLC profiles of Acm_2_-precursor peptide and the reaction solutions after I_2_-oxidation in 50% tetrahydrofuran (THF) at 25–80 °C with a linear gradient from 10 to 40% CH_3_CN in 60 min (0.5%/min) after 10 min of injection.

The ionization of the N-terminal amino group and the carboxyl group at the C-terminus or on side chains, such as Glu8 in ST_h_(6–18), in precursor peptide are affected by the pH of the solvent being used. Therefore, to estimate the pH dependence of topological selection in the folding of ST_a_, we further examined disulfide formation in 50% MeOH at pH 7 and 9. The I_2_-oxidation of the Acm_2_-precursor peptide predominantly yielded the topological isomer as well as in the case at pH 2, although the yields of the topological isomer were lower at both pH 7 and 9 compared to that at pH 2 (Figure 3A). These results suggest that local ionic interactions are not significant for the topological selection of ST_a_ folding during chemical synthesis.

Temperature is also the important factor that affects reaction velocity and the extent of disulfide bond formation during chemical synthesis. The increased reaction temperature increases not only molecular oscillations of a molecule in solution but also the flexibility of the structure of a precursor peptide. To investigate the effects of temperature on I_2_-oxidation, reaction temperatures were varied from 30 to 80 °C. As shown in Figure 3B, the topological isomer was the predominant product by I_2_-oxidation at all temperatures, although the yield of the topological isomer decreased in a temperature-dependent manner and side reactions, including the methylation of carboxyl groups, were clearly observed at temperatures over 50 °C. These results suggest that the conformation of the precursor peptide is tightly restricted by two disulfide bonds.

### 2.3. The Solution Structures of the Topological Isomer and Acm_2_-Precursor Peptide Determined by NMR Spectroscopy

To obtain structural information regarding folding, we carried out NMR measurements of the topological isomer and the Acm_2_-precursor peptide of ST_h_(6–18). NMR measurements of the Acm_2_-precursor peptide were performed in a 50% CD_3_COOD/50% D_2_O or a 50% CD_3_COOH/10% D_2_O mixture at pH 3 to avoid methylation in the acidic methanol solvent that was used for I_2_-oxidation (Figure S4). The peptide conformations related to topological selection were not affected by the types of buffers or solvents, as described above. 

Correlation Spectroscopy (COSY) and Nuclear Overhauser Effect Spectroscopy (NOESY) spectra were acquired and used for proton assignment and to obtain distance constraints. Using the CNS program, 2000 structures were calculated, and 10 structures with the lowest total energy structures were selected, as shown in Figure S6. The overall average root mean square deviation (RMSD) values for the backbone heavy atoms and all heavy atoms, including side chains of topological isomer and the Acm_2_-precursor peptide, are summarized in Table S1. The RMSD value for the ST_a_ molecule was quite low, since the three intra-molecular disulfide bonds make the ST_a_ molecule much more rigid, and therefore, the structural calculation provided such a small RMSD value. The superposition of the structures shows that the calculated structures of the topological isomer clearly converged (Figure S6), suggesting that the structure of the topological isomer is rigidly maintained by virtue of its intra-molecular disulfide bonds as well as that of the native form. On the other hand, the Acm_2_-precursor peptide also showed a well-converged backbone structure, but its side chains, especially the Acm groups at C2 and C5, were relatively disordered, suggesting that the Acm groups might be flexible in this solution (Figure S7). This result was consistent with the observation that the H_N_ of Acm groups could not be defined due to signal broadening.

**Figure 4:**
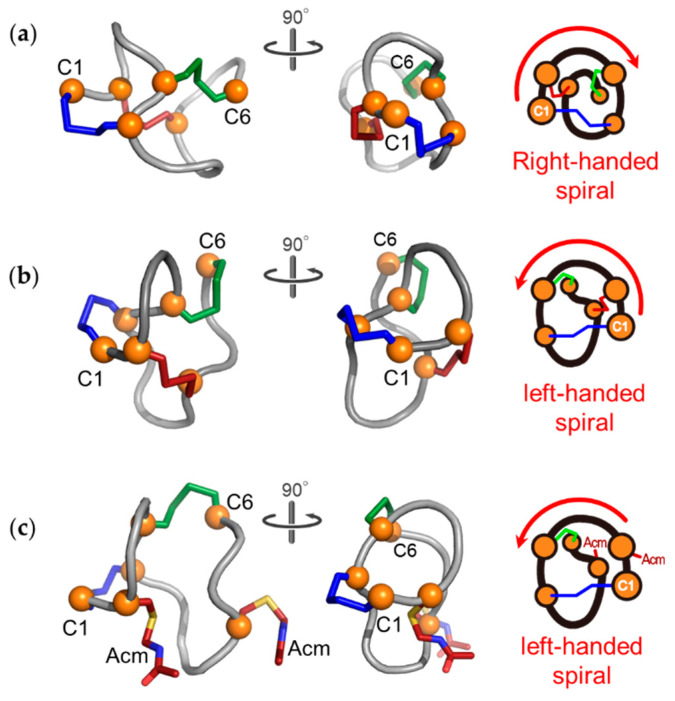
The schematic drawings (*left* and *middle*) of (**a**) the native form, (**b**) topological isomer, and (**c**) Acm_2_-precursor peptide of ST_h_(6–18). Their backbone structures were illustrated by cartoon representations. The *orange spheres* represent the Cα of Cys. The disulfide linkages, C1–C4, C2–C5, and C3–C6, were illustrated by stick representation colored by *blue*, *red*, and *green*, respectively. Schematic drawings of each structure (*right*) showed the right or the left-handed spiral of their backbone when seen from the N- to the C-terminal.

**Figure 5:**
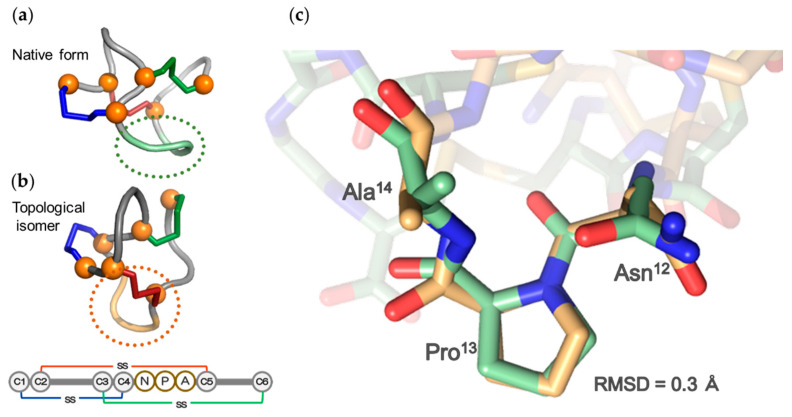
The receptor binding site (-Asn-Pro-Ala-) of ST_a_ was shown in (**a**) the native form and (**b**) topological isomer illustrated by *light green* and *light orange*, respectively. (**c**) The superposition of the receptor binding site of the native form and the topological isomer.

The structure of the native form of ST_a_ was determined by X-ray crystallography in a previous study [16]. Based on the reported structure, the backbone of the native form adopts a right-handed spiral when seen from the N- to the C-terminal (Figure 4A). In contrast, the structure of the topological isomer of ST_h_(6–18) adopted a left-handed spiral backbone structure that was dramatically different from that of the native form, as shown in Figure 4B. The native form of ST_a_ consists of three β-turn moieties of which Cys7(C2)–Cys10(C3) and Asn12–Cys15(C5) form the type I β-turns and Cys15(C5)–Cys18(C6) forms the type II β-turn [16]. However, the topological isomer possessed only one β-turn (type I) in the Asn12–Cys15(C5) region, as shown in Figure 5. Importantly, the structure of this region, corresponding to the receptor binding site, of the topological isomer, was similar to that of the native type ST_a_, indicating that the biologically active conformation is still maintained in the structure of the topological peptide.

The NMR measurements revealed that the Acm_2_-precursor peptide also showed that the molecule has a left-handed spiral backbone structure, similar to the topological isomer (Figure 4C). These results were consistent with previously reported results, showing that the CD spectra of the Acm_2_-precursor peptide were similar to that of the topological isomer of ST_p_(5–17) (Figure S5) [14]. The Asn12–Ala14 region in the Acm_2_-precursor molecule formed a γ-turn structure. The topological isomer contains only three backbone hydrogen bonds, although the native form of ST_a_ possesses five hydrogen bonds in its backbone structure (Table S2). Two of the three hydrogen bonds in the topological isomer were also retained in the Acm_2_-precursor peptide (Table S2). These results suggest that the left-handed spiral backbone structures of the topological and the Acm_2_-precursor peptides are stabilized, not only by hydrogen bonds, but they are also significantly restricted by the two disulfide bonds.

## 3. Discussion

The regioselective formation of the disulfide-rich peptide ST_a_ produced the topological isomer and regulated the conformation of the peptide. To elucidate the folding mechanism of the topological isomer during the chemical synthesis, the disulfide bonds of ST_a_ peptides were formed by a stepwise method using a combination of air-oxidation and I_2_-oxidation. The Acm_2_-precursor peptide with two disulfide bonds was treated with iodine under several conditions. The recovery of the topological isomer was improved when the I_2_-oxidation was carried out under acidic conditions; however, the topological isomer was exclusively produced from the Acm_2_-precursor peptide regardless of the pH value (Table 1 and Figure 3A). 

It is known that uroguanylin, which is structurally homologous to ST_a_ (Figure 1A), with two disulfide linkages (C2–C5 and C3–C6 connectivity) exhibits conformational switching between the native form and the topological isomer under acidic conditions [17,18] but not at neutral pH. Uroguanylin is sufficiently flexible to undergo a conformational change, and the protonation of carboxylate groups appears to support this conformational exchange. On the other hand, the Acm_2_-precursor peptide with two disulfide linkages (C1–C4 and C3–C6 connectivity) was detected as a single conformer on HPLC (Figure S2B), which may contain sufficient information to allow the topological isomer to be formed irrespective of the pH. In addition, the synthetic yield of the topological isomer decreased in a temperature-dependent manner, but the topological isomer was still obtained predominantly (Figure 3B). Moreover, only the topological isomer was obtained by I_2_-oxidation in the all solvents used in these experiments, as shown in Figure 2. Alcohols with low dielectric constants are widely used as an inducer of α-helical structures and stabilizer of β-turns in peptides due to the artificial enhancement in intra-molecular hydrogen bonds. Our results suggest that the dielectric constant of the solvent or the temperature used had no effect on the topological selection of ST_a_ folding, indicating that the conformation of the Acm_2_-precursor peptide is restricted by two disulfide linkages and/or two Acm groups. This observation is consistent with the results of CD measurements indicating that the spectra of the Acm_2_-precursor peptide were nearly the same when several buffers were used (Figure S5).

The structure of the native form of ST_a_ has been determined by X-ray crystallography [16]. However, sufficient structural information on the topological isomer is lacking. To obtain further insights into the folding of the topological isomer, we determined the structures of the topological isomer and Acm_2_-precursor peptide. As shown in Figure 4, the topological isomer had a clearly different structure from that of the native form in spite of the fact the both structures contained the same disulfide bonds and possessed a left-handed spiral structure, as seen from the N-terminal. These observations suggest that the topological isomer is a kinetically trapped product that is produced during chemical synthesis, and that the regioselective disulfide formation provides a product with a different conformation from the native peptide in spite of the fact that both contain the same disulfide bonds.

The native type of ST_a_ requires a right-handed spiral structure for the folding process. Based on the NMR structure of the Acm_2_-precursor molecule, the C- or N-terminal moieties need to pass through the inside of the Acm_2_-precursor molecule, the structure of which is formed by the backbone structure and intra-molecular disulfide bonds, in order for the dynamic inter-conversion between the left- and right-handed spiral structure to occur. However, it is possible that the side chains of the Asn12 and Thr16 residues that are buried in the cavity in the spiral structure could interrupt the inter-conversion. The Acm group of Cys also interrupts the inter-conversion by virtue of its bulkiness, the size of which is comparable to that of the side chain of an Arg residue, and therefore, steric hindrance could occur between the Acm groups and other side chains, such as Asn12 and Thr16. As reported previously, the native type of ST_a_ is produced as a minor product regardless of the order of the regioselective disulfide bond formation [8]. It has been reported that the precursor peptide, ST_p_(5–17) with two disulfide bonds at C2–C5 and C3–C6, showed two conformers on HPLC, and one conformer and another conformer yielded the topological isomer and the native type ST_p_ peptide, respectively [9]. Combined with this observation and the results in these experiments, it appears that the precursor peptides are kinetically trapped during the chemical synthesis and regulate subsequent disulfide formation at the last step in its folding. In addition, our results also raise an alarm that the synthesis of a disulfide-rich peptide by the regioselective method should not be undertaken unless the native type and topological isomer can be separated [19,20].

The results reported herein demonstrate that the stepwise disulfide formation strategy for Cys-rich peptides can provide not only a native form but also kinetically trapped topological isomer. Importantly, only the topological isomer was obtained when the C2–C5 disulfide bond of ST_a_ was formed in the last step [8], suggesting that the structure of its Acm_2_-precursor peptide and/or the order of disulfide formation leads to the folding of disulfide-rich peptides in the case of a chemical synthesis. Thus, chemical synthesis provides the possibility of preparing not only the native type but also the topological isomer, which still possesses biological activity. This topological isomer promises to provide new strategies for the use of peptides, such as in the preparation of vaccines.

## 4. Materials and Methods

### 4.1. Materials and Apparatus

All chemicals and solvents were reagent grade unless otherwise described. The HPLC apparatus consisted of a Waters M600 multisolvent delivery system (Bedford, MA, USA) equipped with a Hitachi L-3000 detector and a D-2500 chromato-integrator or a LaChrom Elite system equipped with an L-2400 detector and a D-2500 chromato-integrator (Hitachi High-Technologies Corporation, Tokyo, Japan).

### 4.2. Syntheses of Peptide

The synthesis of the protected ST_h_(6–18) peptide was performed manually by the solid-phase method, as described previously [8,9,21]. Briefly, protecting groups for functional groups were used, and the protected peptide resins were treated with anhydrous hydrogen fluoride (HF) in the presence of anisole at 0 °C for 60 min, as previously described [8], to remove the protecting groups except for the Acm groups at given Cys residues (Figure S1) [8]. After drying in vacuo, the resulting residues were air-oxidized in 0.5 M AcONH_4_ buffer (pH 8.0) for two days to randomly form two disulfide bonds. The target peptide with the correct two disulfide bonds and 2 × Cys(Acm) residues, ([Cys(Acm)^7,15^,2SS]-ST_h_(6–18)), was predominantly produced and purified by HPLC, as described below [8].

### 4.3. I_2_-Oxidation of the Peptide with Two Disulfide Bonds and 2 × Cys(Acm)

To investigate the effect of solvent on the folding of ST_a_ in the chemical synthesis, [Cys(Acm)^7,15^,2SS]-ST_h_(6–18) (a precursor peptide with two disulfide bonds) was treated with iodine to form a third disulfide bond under the several sets of conditions, as summarized in Table 1. Briefly, the purified precursor peptide (5 × 10^−4^ M) was dissolved in 20% MeOH or other organic solvent, the resulting solution was dropped into the same volume of 20 × 10^−3^ M I_2_ in 80% MeOH or other organic solvents containing 0.2 M HCl, and it was allowed to stand for 30 min at room temperature [8,9,15]. The reaction was stopped by adding the L-ascorbic acid saturated aqueous solution. The reaction product was directly purified by HPLC, as described below [8].

### 4.4. Reversed-Phase High Performance Liquid Chromatography (RP-HPLC)

Peptides were separated by RP-HPLC using a Cosmosil 5C_18_-AR-II column (4.6 × 150 mm, Nacalai tesque, Inc., Kyoto, Japan), as described below. The synthetic peptides were eluted with a linear gradient of 5–50% or 10–50% CH_3_CN in 0.05% trifluoroacetic acid with increasing concentrations of CH_3_CN of l.0%/min or 0.5%/min at a flow rate of 1.0 mL/min. Fractions of the eluates were monitored for absorbance at 220 nm and confirmed by MALDI-TOF/MS and amino acid analyses.

### 4.5. Matrix-Assisted Laser Desorption/Ionization Time of Flight Mass Spectrometry (MALDI-TOF/MS)

The molecular masses of peptides were determined by means of an AXIMA confidence spectrometer (SHIMADZU Co., Kyoto, Japan) in the positive ion mode. Mass spectrometric analyses of peptides were carried out in the linear or reflector modes using α-cyano-4-hydroxycinnamic acid (Sigma-Aldrich Co., Tokyo, Japan) as a matrix. In a typical run, the lyophilized peptide (ca. 0.1 nmol) was dissolved in 0.05% TFA/50% CH_3_CN (1 µL), mixed with 1 µL of a matrix solution (10 mg/mL), and air-dried on the sample plate for MALDI-TOF/MS.

### 4.6. Amino Acid Analysis

Peptides were treated with 5.7 M HCl (constant boiling) at 110 °C for 24 h in a sealed bottle using a Waters picoTAG^TM^ Workstation (Waters Co., Tokyo, Japan). The resulting hydrolysates were taken to dryness in vacuo and dissolved in 20 mM citrate buffer (pH 2). Amino acid analyses were performed on a Hitachi L-2000 amino acid analyzer (Hitachi Ltd., Tokyo, Japan).

### 4.7. CD Measurement

CD spectra were recorded on a JASCO J820 spectropolarimeter at 25 °C. The peptides were dissolved in 20 mM sodium phosphate buffer (pH 6.5), 50% MeOH/0.1 M HCl, or 50% *i*-PrOH/0.1 M HCl.

### 4.8. NMR Measurement and Structure Calculation

All NMR experiments were performed on a JNM-ECA800 spectrometer (JEOL RESONANCE Inc., Tokyo, Japan) at 25°C. The topological isomer of ST_h_(6–18) was dissolved in 20 mM sodium phosphate buffer prepared with D_2_O or a 90% H_2_O/10% D_2_O mixture at pH 6.5. The precursor peptide, [Cys(Acm)^7,15^, 2SS]-ST_h_(6–18), was dissolved in a 50% CD_3_COOD/50% D_2_O or a 50% CD_3_COOH/10% D_2_O mixture at pH 3. The peptide concentration was adjusted to approximately 5 mM and was placed in a 5-mm NMR tube (Shigemi, Tokyo, Japan) for all NMR experiments. Assignment of proton resonances was achieved by using a series of 1D and 2D spectra, including ^1^H, DQF-COSY and ^1^H-^1^H NOESY. The NOE distance constraints for peptides were derived from ^1^H-^1^H NOESY spectra with mixing times of 200 ms. All structure calculations were performed with the CNS program [22]. Structure optimization and energy minimization were achieved using a simulated annealing algorithm. The final 10 lowest energy structures were analyzed using the MOLMOL [23] and PROCHECK programs [24]. Structural statistics for the 10 structures are included in Table S1. Graphical representations were prepared using PyMOL (www.pymol.org). All of these structures have been deposited in the Protein Data Bank (PDB) (http://www.rcsb.org/pdb/) under the accession code 7CSS and 7D37.

## Figures and Tables

**Table 1:** Recoveries for the I_2_-oxidation of the Acm_2_-precursor peptide.

Organic Solvents	pH	Temperature	Recovery (%) *^a^*
50% AcOH	3	25	82
50% MeOH/0.1 M HCl	2	25	74
50% MeOH	7	25	30
50% MeOH/0.1 M NH_3_ aq	9	25	28
50% *i*-PrOH/0.1 M HCl	2	25	63
80% *i*-PrOH/0.1 M HCl	2	25	62
50% THF/0.05% TFA	2	25	71
50% THF/0.05% TFA	2	30	69
50% THF/0.05% TFA	2	40	64
50% THF/0.05% TFA	2	50	54
50% THF/0.05% TFA	2	60	36
50% THF/0.05% TFA	2	70	25
50% THF/0.05% TFA	2	80	19

*^a^* The Acm_2_-precursor peptide, [Cys(Acm)^7,15^,2SS]-ST_h_(6–18), was treated with iodine under various conditions. The recoveries of the topological isomer were determined by HPLC analysis.

## Supplementary Materials

Figure S1: Scheme for synthesis of the topological isomer of ST_h_(6–18) by the stepwise formation of disulfide bonds. Figure S2: RP-HPLC profiles of the reaction solutions at each step of stepwise regioselective formation of disulfide bonds. Figure S3: RP-HPLC profiles of the Acm_2_-precursor peptide after air-oxidation. Figure S4: RP-HPLC profiles of Acm_2_-precursor peptide incubated under several conditions. Figure S5: CD spectra of the native form, topological isomer, and Acm_2_-precursor peptide of ST_h_(6–18). Figure S6: The superpositions of 10 lowest energy structures of the topological isomer and Acm_2_-precursor peptide of ST_h_(6–18). Figure S7: The superpositions of 10 lowest energy backbone structures of Acm_2_-precursor peptide of ST_h_(6–18). Table S1: Data collection and refinement statistics. Table S2: Hydrogen bonds in the backbone structure of the native form, topological isomer and Acm_2_-precursor peptide.

## Abbreviation

2SS: two disulfide bonds; Acm, acetamidomethyl; Acm_2_-precursor, [Cys(Acm)^7,15^,2SS]-ST_h_(6–18); CD, circular dichroism; COSY, correlation spectroscopy; GC-C, guanylyl cyclase-C; HF, hydrogen fluoride; NOESY, nuclear overhauser effect spectroscopy; RMSD, root mean square deviation; ST_a_, heat-stable enterotoxin; THF, tetrahydrofuran.
