# Supplementary material for: Topological Regulation of the Bioactive Conformation of a Disulfide-Rich Peptide, Heat-Stable Enterotoxin
Source: Molecules. 2020 Oct 21;25(20):4798. doi: 10.3390/molecules25204798 (PMC7587965; doi:10.3390/molecules25204798)
Supplement: Supplementary file 1 [file molecules-25-04798-s001.pdf]

Supplementary Materials

Table S1. Data collection and refinement statistics.

No. of restraints	Topological isomer	Acm_2-precursor
All	64	144
NOE distance restraints	61	142
Intraresidue	21	91
Sequential	26	42
Medium range	7	3
Long range	7	6
Disulfide bond	3	2
Deviations from idealized covalent geometry		
Bonds(Å)	0.0074 ± 1.83e-4	0.0134 ± 3.53e-4
Angle(°)	0.718 ± 3.16e-4	1.98 ± 2.97e-2
Impropers(°)	0.371 ± 8.76e-4	0.786 ± 1.07e-2
Mean coordinate RMSD from mean structure[a]		
Backbone heavy atoms	0.01 ± 0.01 Å	0.41 ± 0.16 Å
All heavy atoms	0.29 ± 0.17 Å	1.41 ± 0.28 Å

[a] Root mean square deviation (RMSD) was calculated using 10 possible structures.

Table S2. Hydrogen bonds in the backbone structure of the native form, topological isomer and Acm_2-precursor peptide.

	Hydogen bond			structure
ST_h(6–18)	donor [a]		acceptor [a]	
Native form	Leu9(H_N)	↔	Cys6(CO)	
	Cys10(H_N)	↔	Cys7(CO)	Type I β-turn
	Cys11(H_N)	↔	Cys6(CO)	
	Cys15(H_N)	↔	Asn12(CO)	Type I β-turn
	Cys18(H_N)	↔	Cys15(CO)	Type II β-turn
Topological isomer	Cys7(H_N)	↔	Cys10(CO)	
	Cys11(H_N)	↔	Gly17(CO)	
	Cys15(H_N)	↔	Asn12(CO)	Type I β-turn
Acm_2-precursor	Cys7(H_N)	↔	Cys10(CO)	
	Ala14(H_N)	↔	Asn12(CO)	γ-turn

[a] The structural information about hydrogen bonds in the native form of ST_h(6–18) were based on the X-ray structure of [Mpr[5]]-ST_p(5–17). The residue numbers of ST_p(5–17) were adjusted to those of ST_h(6–18).

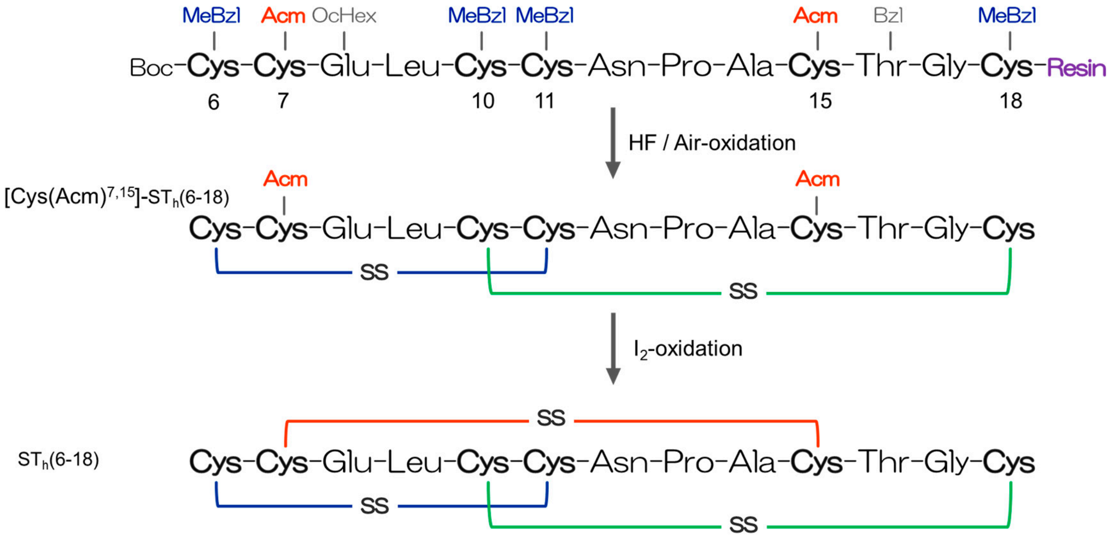

Figure S1. Scheme for synthesis of the topological isomer of ST_h(6–18) by the stepwise formation of disulfide bonds.

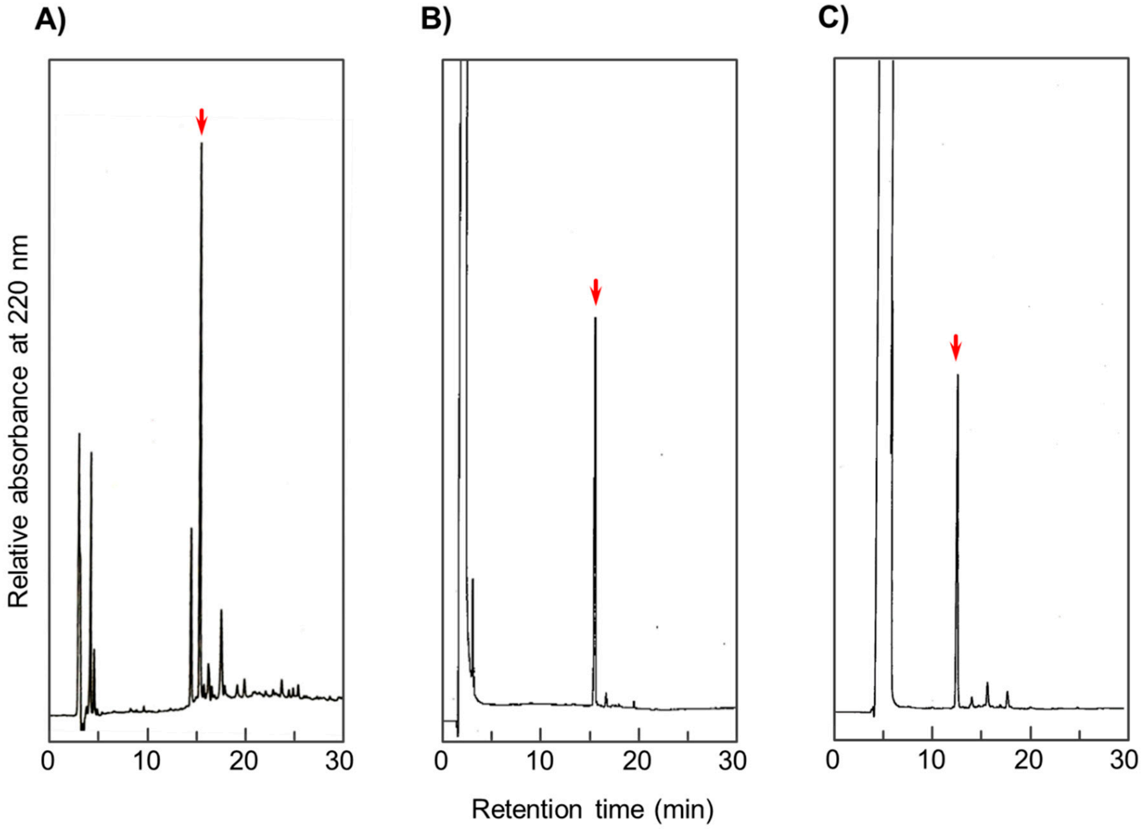

Figure S2. RP-HPLC profiles of the reaction solutions at each step of stepwise regioselective formation of disulfide bonds with linear gradient from 10 to 50% CH_3CN in 40 min (1.0%/min). (A) After air-oxidation of the deprotected peptide in the first step, analytical HPLC of Acm_2-peptides showed two major peaks. The arrow indicates the Acm_2-precursor peptide used for following I_2-oxidation. (B) Re-chromatogram of the peak fraction of Acm_2-precursor peptide in Fig. S2A. (C) I_2-oxidation under the ordinary condition using 50% MeOH produced the topological isomer.

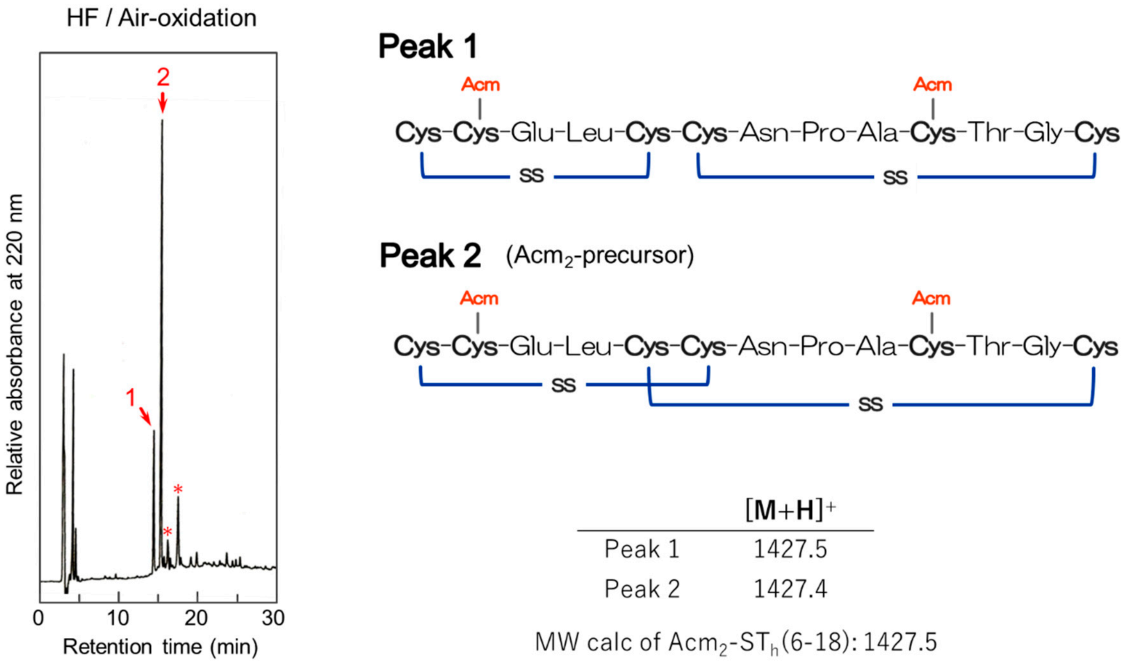

Figure S3. RP-HPLC profiles of the Acm2-precursor peptide after air-oxidation. The peak 1 indicates the fraction contained the Acm2-peptide with C1-C3 and C4-C6 connectivity. The peak 2 indicates the fraction containing the Acm2-precursor peptide. The asterisks indicate the impurities derived from the reagents used for the reaction (Shimonishi Y. et al., *FEBS Lett* **1987,** 215, (1), 165–70).

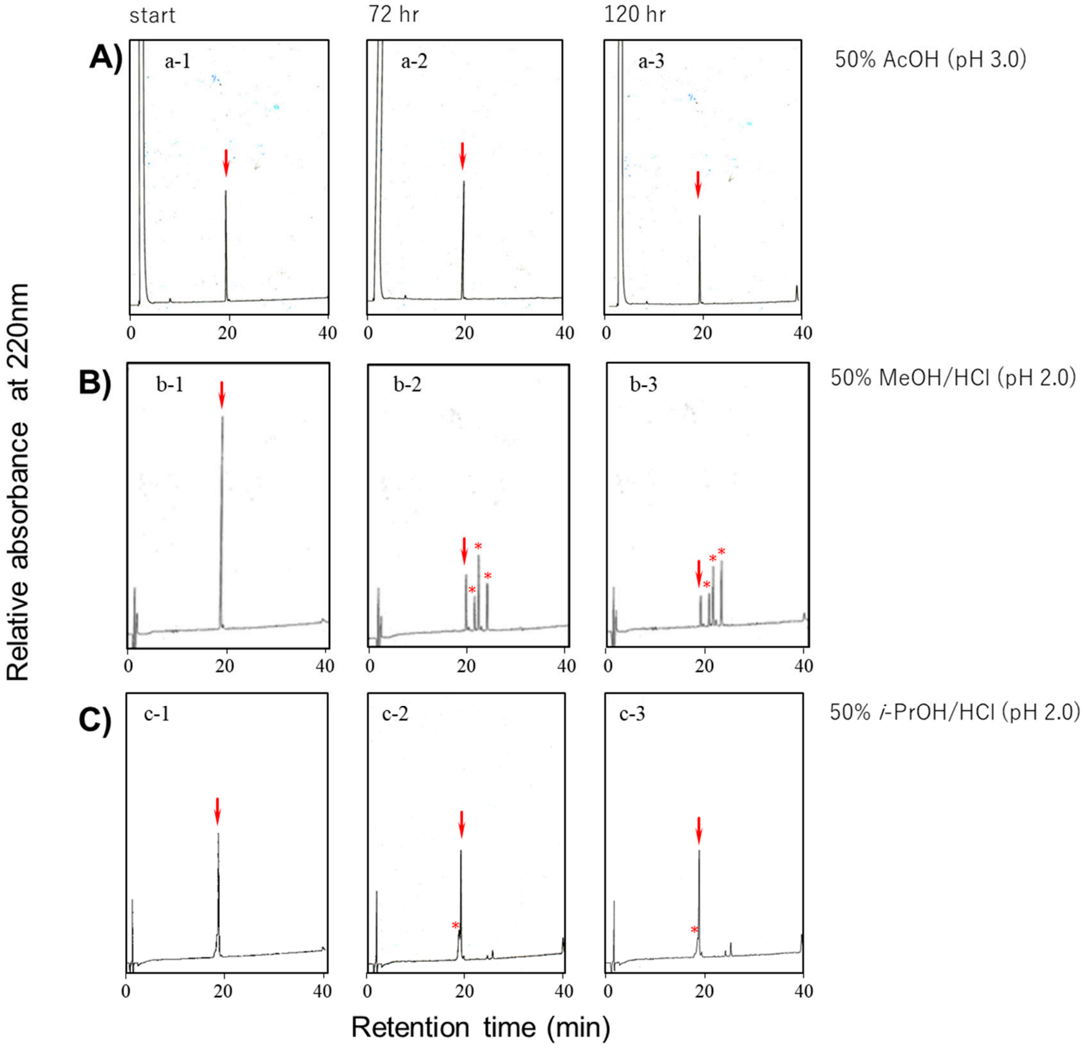

Figure S4. RP-HPLC profiles of Acm2-precursor peptide incubated in (A) 50% AcOH, (B) 50% MeOH/0.1 M HCl, and (C) 50% *i*-PrOH/0.1 M HCl at 25°C for 0, 72, and 120 hr, respectively.

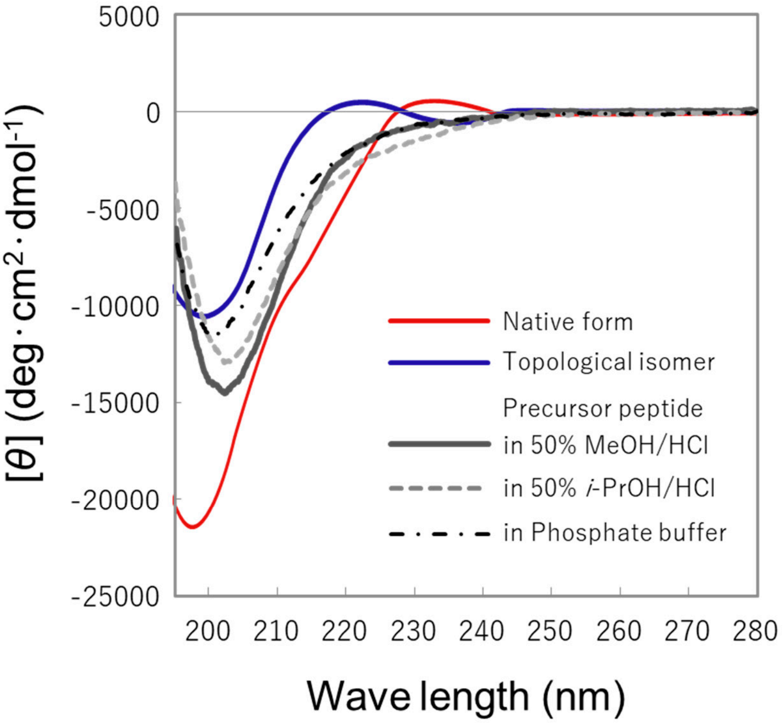

Figure S5. CD spectra of the native form (*solid red line*) and topological isomer (*solid blue line*) of ST_h(6–18) in 20 mM sodium phosphate buffer (pH 6.5). CD spectra of Acm_2-precursor peptide in 50% MeOH/0.1 M HCl (*solid line*), 50% *i*-PrOH/0.1 M HCl (*dashed line*), and 20 mM sodium phosphate buffer (pH 6.5) (*chain line*) at 25°C.

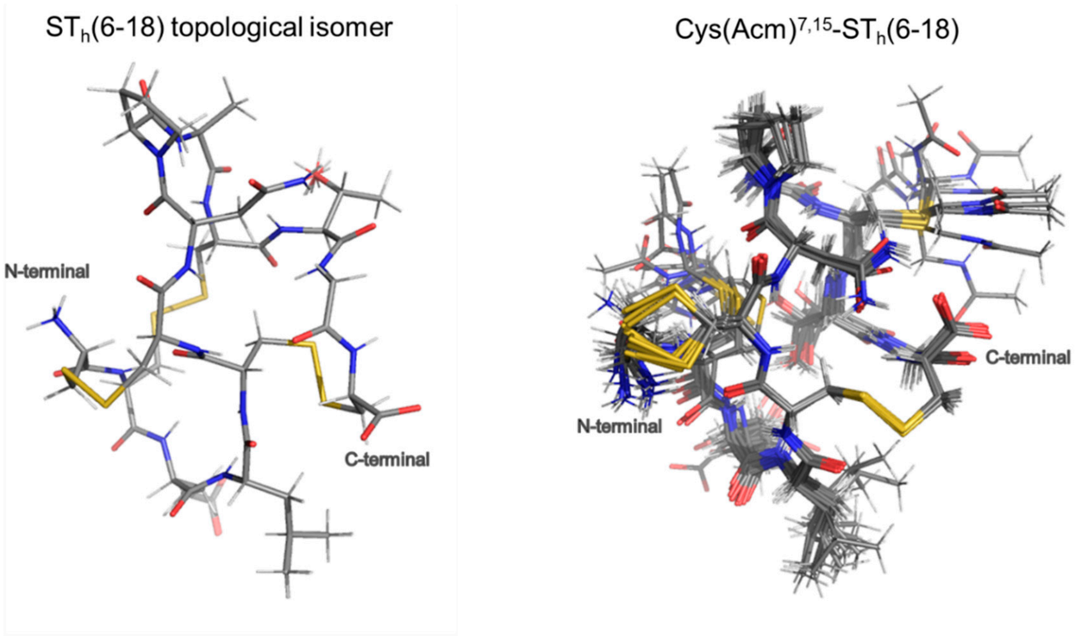

Figure S6. The superpositions of 10 lowest energy structures of (A) the topological isomer and (B) Acm_2-precursor peptide of ST_h(6–18).

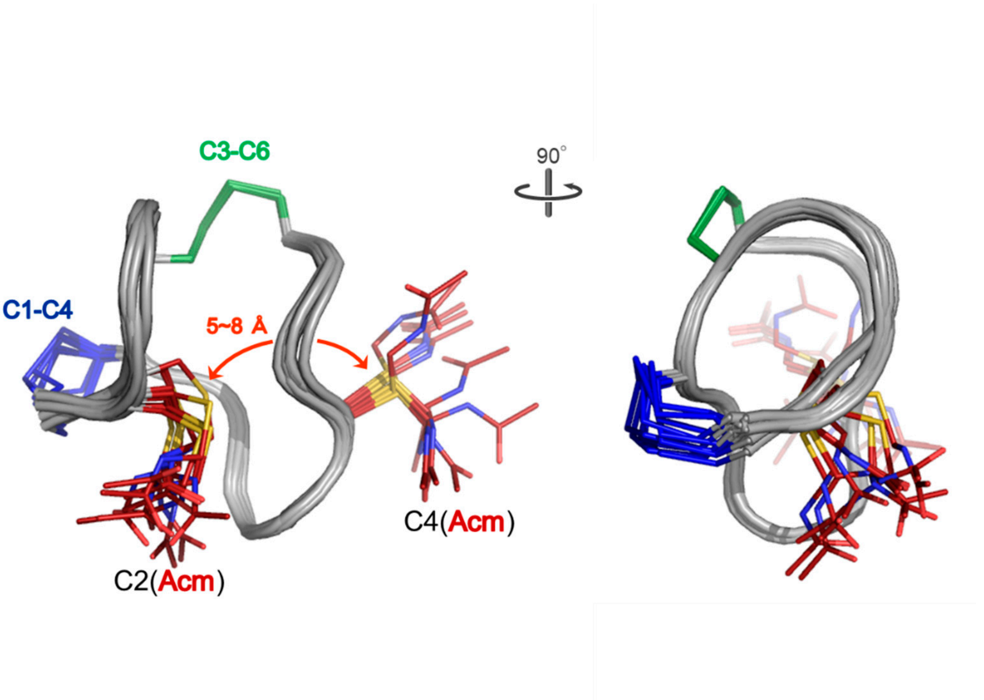

Figure S7. The superpositions of 10 lowest energy structures of Acm_2-precursor peptide of ST_h(6–18). The backbone structures were illustrated by cartoon representation, and the two disulfide bonds and two Cys(Acm) residues were illustrated by stick representation. The C1-C4, C3-C6 linkage, and two Cys(Acm) residues were colored by *blue*, *green* and *red*, respectively.
